# Promotion of behavioural change for health in a heterogeneous population

**DOI:** 10.2471/BLT.20.285227

**Published:** 2021-09-30

**Authors:** Robin Schimmelpfennig, Sonja Vogt, Sönke Ehret, Charles Efferson

**Affiliations:** a Faculty of Business and Economics, University of Lausanne, Internef, CH-1015 Lausanne, Switzerland.

## Abstract

Public health policy often involves implementing cost-efficient, large-scale interventions. When mandating or forbidding a specific behaviour is not permissible, public health professionals may draw on behaviour change interventions to achieve socially beneficial policy objectives. Interventions can have two main effects: (i) a direct effect on people initially targeted by the intervention; and (ii) an indirect effect mediated by social influence and by the observation of other people’s behaviour. However, people’s attitudes and beliefs can differ markedly throughout the population, with the result that these two effects can interact to produce unexpected, unhelpful and counterintuitive consequences. Public health professionals need to understand this interaction better. This paper illustrates the key principles of this interaction by examining two important areas of public health policy: tobacco smoking and vaccination. The example of antismoking campaigns shows when and how public health professionals can amplify the effects of a behaviour change intervention by taking advantage of the indirect pathway. The example of vaccination campaigns illustrates how underlying incentive structures, particularly anticoordination incentives, can interfere with the indirect effect of an intervention and stall efforts to scale up its implementation. Recommendations are presented on how public health professionals can maximize the total effect of behaviour change interventions in heterogeneous populations based on these concepts and examples.

## Résumé

Les politiques de santé publique impliquent souvent l'organisation de campagnes rentables à grande échelle. Lorsqu'il est impossible d'imposer ou d'interdire certains comportements, les professionnels de la santé publique ont parfois recours à des actions induisant un changement de comportement afin d'atteindre des objectifs bénéfiques pour la société. Ces actions sont susceptibles d'entraîner deux effets: (i) un effet direct sur les personnes initialement visées par la campagne; et (ii) un effet indirect provoqué par la pression sociale et l'observation du comportement d'autres personnes. Néanmoins, les attitudes et croyances peuvent considérablement varier au sein de la population; ainsi, ces deux effets peuvent interagir et avoir des conséquences imprévues, inefficaces et contre-intuitives. Les professionnels de la santé publique ont besoin de mieux comprendre cette interaction. Le présent document en illustre donc les principes majeurs en examinant deux domaines clés des politiques de santé publique: le tabagisme et la vaccination. L'exemple des campagnes antitabac montre quand et comment les acteurs de la santé publique peuvent accentuer l'impact d'une action destinée à faire évoluer les comportements en optant pour l'approche indirecte. L'exemple des campagnes de vaccination met en lumière la manière dont les structures d'incitation sous-jacentes, en particulier celles favorisant l'anticoordination, peuvent interférer avec l'effet indirect d'une action et anéantir les efforts déployés pour la mettre en œuvre. Plusieurs recommandations sont formulées afin d'aider les professionnels de la santé publique à amplifier l'effet global des actions de changement comportemental au sein d'une population hétérogène, en s'appuyant sur ces concepts et exemples.

## Resumen

La política de salud pública suele incluir la aplicación de intervenciones rentables y a gran escala. Cuando no es posible imponer o prohibir un comportamiento específico, los profesionales de la salud pública pueden recurrir a intervenciones de cambio de comportamiento para lograr objetivos políticos que sean favorables para la sociedad. Es posible que las intervenciones generen dos efectos principales: i) un efecto directo sobre las personas a las que en principio se dirige la intervención; y ii) un efecto indirecto mediado por la influencia social y por la observación del comportamiento de otras personas. Sin embargo, las actitudes y creencias de las personas pueden ser muy diferentes en toda la población, por lo que estos dos efectos pueden interactuar y producir consecuencias inesperadas, poco útiles y contraproducentes. Los profesionales de la salud pública deben comprender mejor esta interacción. Este documento explica los principios clave de esta interacción al analizar dos áreas importantes de la política de salud pública: el tabaquismo y la vacunación. El ejemplo de las campañas antitabaco muestra cuándo y cómo los profesionales de la salud pública pueden aumentar los efectos de una intervención de cambio de comportamiento si se aprovecha el procedimiento indirecto. El ejemplo de las campañas de vacunación explica cómo las estructuras subyacentes de incentivos, en particular los incentivos de descoordinación, pueden interferir con el efecto indirecto de una intervención y detener los esfuerzos para ampliar su aplicación. A partir de estos conceptos y ejemplos, se formulan recomendaciones sobre cómo los profesionales de la salud pública pueden maximizar el efecto total de las intervenciones de cambio de comportamiento en poblaciones heterogéneas.

## ملخص

غالبًا ما تتضمن سياسة الصحة العامة تنفيذ تدخلات واسعة النطاق ومعقولة التكلفة. عندما لا يكون من المسموح به فرض أو حظر سلوك معين، فقد يعتمد أخصائيو الصحة العامة على تدخلات تغيير السلوك لتحقيق أهداف مفيدة اجتماعيًا للسياسة. يمكن أن يكون للتدخلات تأثيران رئيسيان: (1) تأثير مباشر على الأشخاص المستهدفين مبدئيًا بواسطة التدخل؛ و(2) تأثير غير مباشر يتوسطه التأثير الاجتماعي ومراقبة سلوك الأشخاص الآخرين. ومع ذلك، فإنه يمكن أن تختلف مواقف الأشخاص ومعتقداتهم بشكل ملحوظ بين فئات السكان، مما يؤدي إلى أن هذين التأثيرين يمكن أن يتفاعلا لينتجا تبعات غير متوقعة وغير مفيدة وغير بديهية. يحتاج أخصائيو الصحة العامة إلى فهم هذا التفاعل بشكل أفضل. توضح هذه الورقة المبادئ الرئيسية لهذا التفاعل من خلال فحص مجالين مهمين لسياسة الصحة العامة: تدخين التبغ والتطعيم. إن مثال حملات مكافحة التدخين يوضح متى وكيف يمكن لأخصائيي الصحة العامة تعظيم تأثيرات تدخل التغيير السلوكي من خلال الاستفادة من المسار غير المباشر. يوضح مثال حملات التطعيم كيف يمكن لهياكل الحوافز الأساسية، وخاصة حوافز منع التنسيق، أن تتداخل مع التأثير غير المباشر للتدخل، وتعطل الجهود المبذولة لتوسيع نطاق تنفيذه. يتم تقديم توصيات حول كيف يمكن لأخصائيي الصحة العامة تعظيم التأثير الكلي لتدخلات التغيير السلوكي في مجموعات سكانية غير متجانسة، بناءً على هذه المفاهيم والأمثلة.

## 摘要

公共卫生政策通常涉及实施具有成本效益的大规模干预措施。当不允许强制实施或禁止特定行为时,公共卫生专业人员可以利用行为改变干预措施来实现对社会有益的政策目标。干预措施可以产生两个主要影响:(i) 对干预措施最初针对的人群的直接影响;以及 (ii) 由社会影响和观察他人行为产生的间接影响。然而,人们的态度和信念在整个人群中可能存在显著差异,导致这两种影响会相互作用,产生意想不到的、无益的和违反常理的后果。公共卫生专业人员需要更好地理解这种相互作用。本文通过研究公共卫生政策的两个重要领域(吸烟和接种疫苗),说明了这种相互作用的关键原则。反吸烟运动的事例表明,公共卫生专业人员可以在何时以及通过何种方式利用间接途径来增强行为改变干预措施的效果。疫苗接种运动的事例表明,潜在诱因结构(特别是反协同诱因)如何干扰干预的间接影响和阻止扩大干预的实施范围。基于以上概念和事例,我们针对公共卫生专业人员可以如何在异质群体中最大程度上提高行为改变干预措施的总体效果,提出了建议。

## Резюме

Политика в области общественного здравоохранения часто включает в себя внедрение рентабельных крупномасштабных мероприятий. Когда предписывать или запрещать определенное поведение недопустимо, специалисты в области общественного здравоохранения могут прибегнуть к мероприятиям по изменению поведения для достижения целей социально-ориентированной политики. Мероприятия могут иметь два основных эффекта: (i) прямое воздействие на людей, которые изначально являются объектом мероприятия; и (ii) косвенное воздействие, опосредованное влиянием социальной среды и наблюдением за поведением других людей. Однако взгляды и убеждения людей могут заметно различаться среди населения, в результате чего эти два эффекта могут взаимодействовать, приводя к неожиданным, бесполезным и нелогичным последствиям. Специалистам в области общественного здравоохранения необходимо более тщательно изучать и понимать такое взаимодействие. Данный документ иллюстрирует ключевые принципы такого взаимодействия, исследуя две важные области политики в области общественного здравоохранения: курение табака и вакцинацию. Пример кампаний по борьбе с курением показывает, когда и как специалисты в области общественного здравоохранения могут усилить эффект мероприятия по изменению поведения, воспользовавшись косвенным путем. Пример кампаний по вакцинации показывает, как основные структуры стимулирования, особенно антикоординационного стимулирования, могут помешать косвенному эффекту воздействия и сорвать попытки по расширению его внедрения. На основе этих концепций и примеров представлены рекомендации о том, как специалисты в области общественного здравоохранения могут усилить общий эффект мероприятий по изменению поведения в неоднородной популяции населения.

## Introduction

Public health policy objectives often conflict with local culture.^1^^–^^3^ Thus, to avoid a backlash when trying to change people’s behaviour, policy-makers may often resist policies that forbid or mandate a specific behaviour. They may instead work with public health professionals to design interventions aimed at changing behaviour that do not restrict freedom of choice.^4^ However, for various reasons, such as budgetary or infrastructure constraints, it is often impractical to ensure that the entire population is exposed to such interventions. One workaround is to tap into social influence mechanisms. Social influence can help bring about behaviour change in individuals in the population who cannot be reached by the intervention. When individuals have an interest in behaving like those around them, the behaviour change among those initially exposed to the intervention can spill over to influence those not exposed.^4^^–^^7^ Consequently, the potential of social influence and behavioural spillover to amplify the impact of policy interventions in this way has received much attention in various areas of public health, such as scaling up vaccine delivery,^8^^,^^9^ reducing the prevalence of female genital mutilation,^1^ combating gun violence and smoking,^10^^,^^11^ and challenging norms on sex-selective abortion.^2^^,^^12^

When socially beneficial interventions are optimally designed and implemented, behavioural spillover can dramatically amplify their effects in a cost-efficient way. The mechanisms that drive spillover, however, will be subtle and will vary according to culture, the group targeted by the intervention and the behaviour of interest.^13^^,^^14^ Here we examine two subtle mechanisms that may help public health professionals maximize behaviour change in support of public health objectives.^15^^,^^16^ First, we draw on the results of empirically informed models to show how ordinary forms of heterogeneity in attitudes, beliefs, and behaviours between individuals can determine which segment of the population should be targeted by an intervention. Second, we examine how behavioural spillover can be critically influenced by the interaction between two common forms of heterogeneity: (i) the variation in when people respond to others changing their behaviour (i.e. the variation in people’s tendency to make a socially beneficial behavioural choice based on how common this choice is among their peers); and (ii) the variation in perceptions of the costs and benefits associated with a behavioural choice.

Here we consider antismoking and vaccination campaigns to illustrate how these two common forms of heterogeneity can interact to affect behaviour change in unexpected ways. Current discussions of social influence in behavioural public health policy do not typically account for these important issues.^8^^,^^9^^,^^17^ We also provide recommendations on managing the trade-offs involved in attempting to change behaviour when people have differing attitudes and beliefs but still influence each other.

## Behavioural spillover

**Fig. 1:**
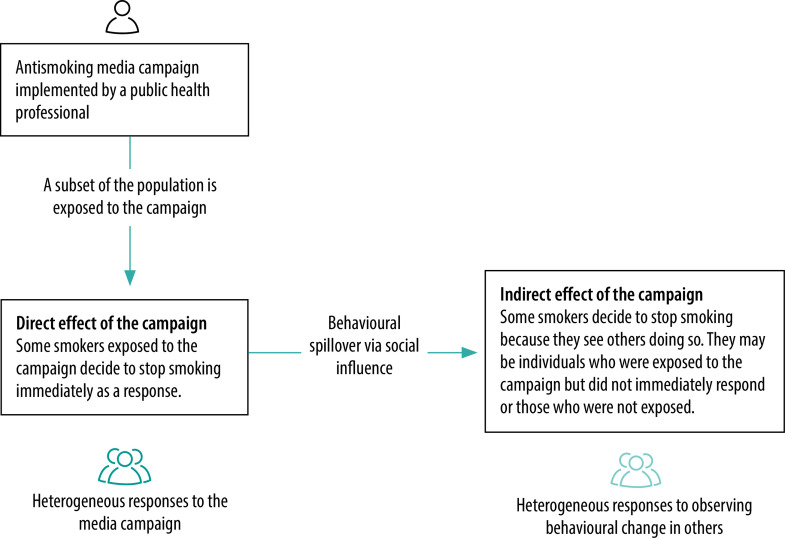
Direct and indirect effects of an antismoking campaign

Note: The total effect of a campaign is the sum of its direct and indirect effects.

To conceptualize how social influence can affect behavioural spillover, consider the example of a public health professional who wants to maximize behavioural change among smokers. The professional will have an intervention in mind, say a media campaign to persuade people to stop smoking,^11^^,^^18^^,^^19^ which could target a subset of the population. We can split the total effect of the campaign into a direct effect and an indirect effect (Fig. 1). Some people in the population will change their behaviour after being directly exposed to the media campaign (i.e. the direct effect). The remainder will not have changed their behaviour, either because they did not respond after being exposed to the campaign or because they were not exposed to it. Crucially, this second part of the population may still change their behaviour by stopping smoking after observing others doing so. Behaviour change brought about by social influence in this way is the indirect effect of the intervention. Research has shown that, under some conditions, the indirect effect can be more pronounced than the initial direct effect.^5^^,^^7^

Individuals differ from each other for many reasons. For example, they may differ in their tendency to respond to an intervention,^20^ which will shape the direct effect of the intervention. A lifelong smoker who has never considered stopping will be much less likely to be persuaded by a media campaign than someone who just started smoking. Alternatively, individuals may differ in when they respond to social information, which will influence the indirect effect of the intervention. Some people may only stop smoking after observing 50% of people they know doing so, others may change their behaviour after only 20% have stopped and others only when 85% have done so. Some public health professionals may regard such mundane forms of heterogeneity in attitudes and preferences as secondary details, but they are not. The key question is how they interact to shape behavioural spillover and, by extension, influence the total effect of the behaviour change intervention. The answer to this question involves resolving the fundamental trade-off between maximizing the direct or indirect effect of an intervention.

## Targeting amenable or resistant individuals

In our example on modifying smoking behaviour, the public health professional must maximize the total effect of the policy initiative by taking advantage of social influence and associated behavioural spillover. Accordingly, a basic decision needs to be made. Assuming the whole population cannot be reached (e.g. due to budgetary constraints), the health professional must decide how many people should be targeted by the media campaign and who these people should be. Given that everyone in the target population smokes, intuition might suggest prioritizing those more amenable to change. In fact, targeting amenable individuals has often been explicitly or implicitly recommended.^1^^,^^21^^,^^22^ Moreover, even if individuals amenable to change are not specifically targeted, they may self-select to be exposed to the intervention precisely because they find the policy objective attractive. In essence, smokers uncomfortable with their behaviour may seek out information encouraging them to stop smoking, which will increase the direct effect of antismoking media campaigns. 

The intuitive appeal of targeting amenable individuals is unreliable because the heterogeneity in attitudes and preferences throughout the population creates a fundamental but poorly understood trade-off for public health professionals.^13^ To illustrate the nature of this trade-off, consider two extreme strategies: (i) targeting the segment of the population most amenable to change and most likely to respond to the intervention; and (ii) targeting the most resistant segment. There is also the intermediate case of targeting randomly selected individuals. The main characteristics of this trade-off are summarized in Table 1. The best way to resolve the trade-off, and thus maximize the total effect of an intervention, will depend both on how common different attitudes are in the population and on how people respond to the intervention and to other people’s behaviour change. 

**Table 1:** The trade-off between targeting amenable or resistant individuals with behavioural interventions

Subset of population targeted by the intervention	Effect of the intervention	
	Direct effect	Indirect effect
**Amenable individuals**	Targeting those most likely to change behaviour maximizes the direct effect of the intervention	Targeting those most likely to change behaviour means relying on the indirect effect of the intervention to influence those least likely to change. For a given magnitude of direct effect, targeting those most likely to change minimizes the indirect effect of the intervention and, by extension, behavioural spillover
**Resistant individuals**	Targeting those least likely to change behaviour minimizes the direct effect of the intervention	For a given magnitude of direct effect, targeting those least likely to change behaviour maximizes the indirect effect of the intervention because those most likely to change will be affected indirectly but were not directly targeted
**Randomly selected individuals**	The size of the direct effect will be intermediate between the effects of targeting either amenable or resistant individuals	The size of the indirect effect will be intermediate between the effects of targeting either amenable or resistant individuals

To gain an insight into which strategy may be preferable in a particular situation, we have adapted Efferson et al.’s empirically grounded modelling work.^13^ An individual’s pre-existing attitudes will shape both how likely the individual is to respond to exposure to a media campaign and how many other people have to stop smoking before the decision is made to do so. Pre-existing attitudes in a population vary on a continuous scale from relatively amenable to change to resistant to change. Furthermore, attitudes are not fixed but can evolve, for example, after being exposed to a public health professional’s media campaign.

**Fig. 2:**
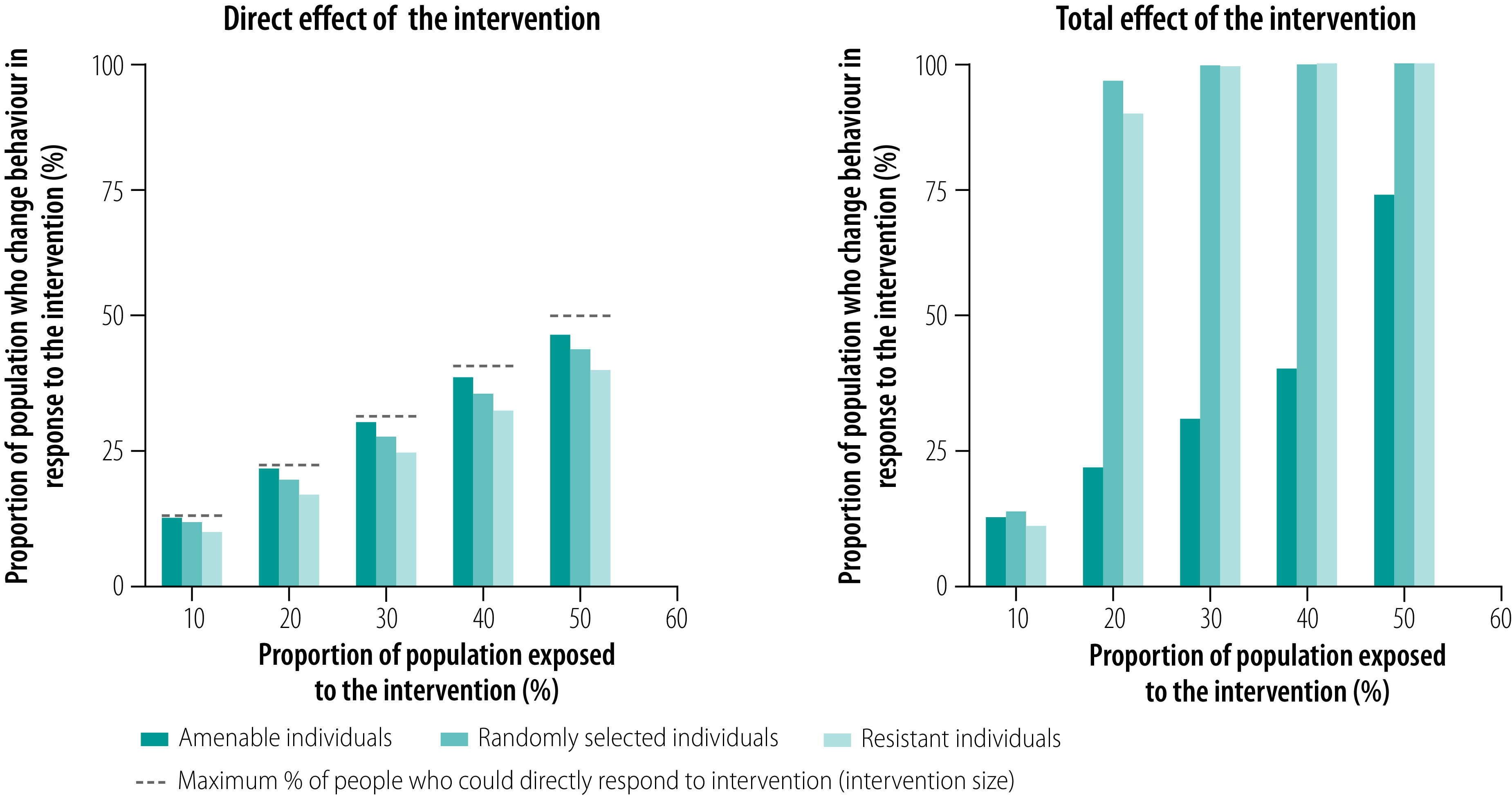
Direct and total effects of a behavioural change intervention with the assumption that the probability of change is high except in people most resistant to change, by target group

Note: The total effect of the intervention is the sum of its direct and indirect effects. People’s responses to the intervention are affected by their pre-existing attitudes, specifically by how resistant or amenable they are to the new behaviour. For this figure, it was assumed that the probability of behaviour change on exposure to the intervention was relatively high for all but the most resistant. The target group comprises the type of individuals who would initially be targeted by the intervention. For example, if the target size is 50% and the target group comprises resistant individuals, the 50% of the population with the highest resistance (based on their attitudes) is targeted. More details are available from the data repository.^23^

**Fig. 3:**
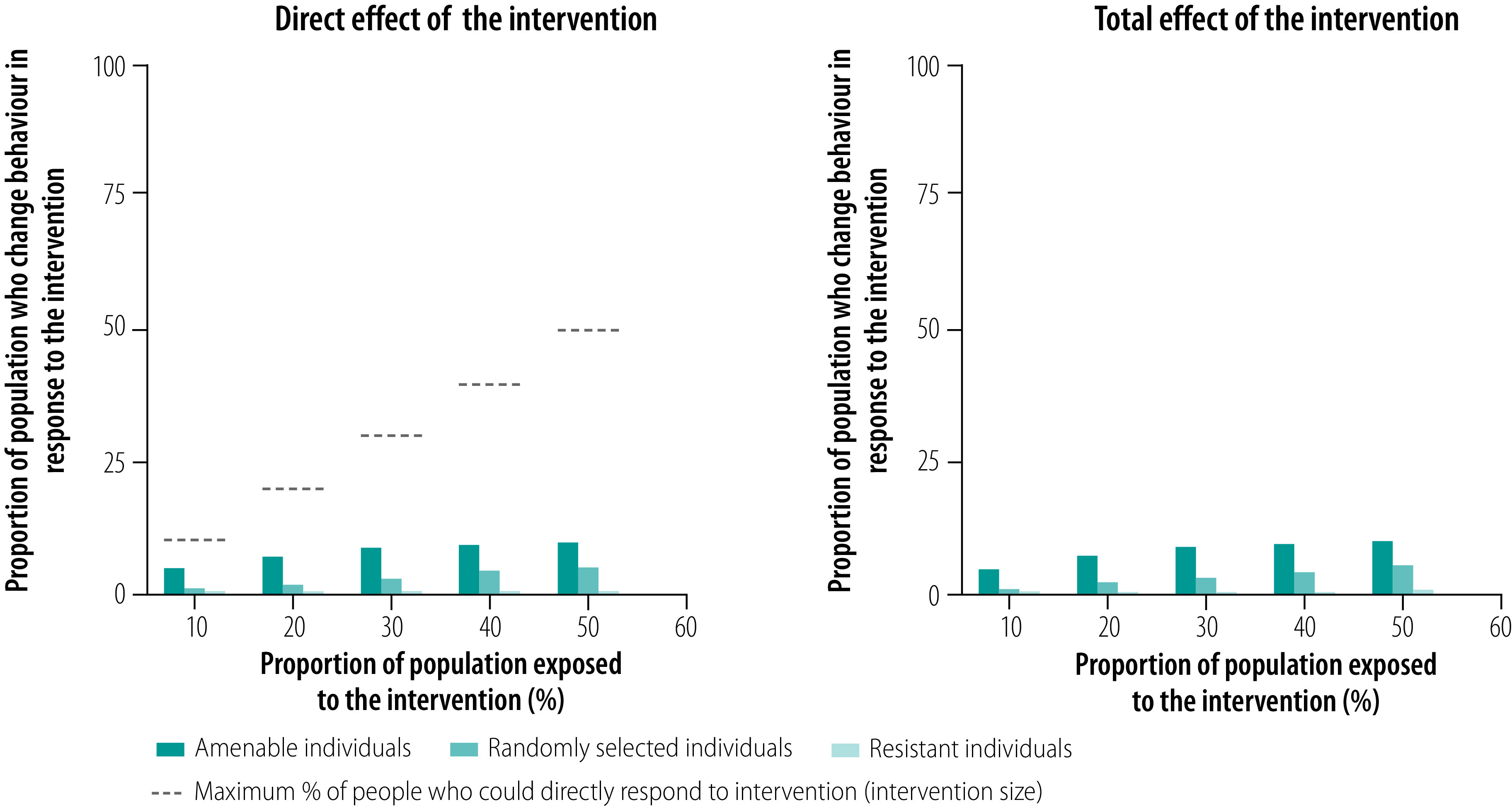
Direct and total effects of a behavioural change intervention with the assumption that the probability of change is low except in people most amenable to change, by target group

Note: The total effect of the intervention is the sum of its direct and indirect effects. People’s responses to the intervention are affected by their pre-existing attitudes, specifically by how resistant or amenable they are to the new behaviour. For this figure, it was assumed that the probability of behaviour change on exposure to the intervention was relatively low for all but the most amenable. The target group comprises the type of individuals who would initially be targeted by the intervention. For example, if the target size is 50% and the target group comprises resistant individuals, the 50% of the population with the highest resistance (based on their attitudes) is targeted. More details are available from the data repository.^23^

Fig. 2 and Fig. 3 illustrate how the direct and total effects of a behavioural intervention (e.g. an antismoking campaign) can change as the proportion of the population targeted increases and as different types of individual are targeted in two contexts: where the probability of change is high or low, respectively. When the probability of responding to an intervention by changing behaviour is high for everyone except those most resistant to change (Fig. 2), all individuals but the most resistant will probably change their behaviour and stop smoking if exposed to the media campaign. In this case, targeting individuals amenable to change would yield only a small increase in the direct effect of the intervention and targeting either randomly selected people or resistant people would yield even smaller increases. Surprisingly, behavioural spillover and, by extension, the total effect of the intervention are dramatically larger when either randomly selected or resistant people are targeted than when amenable people are targeted. In this situation, the indirect effect dominates the direct effect and targeting amenable people minimizes the indirect effect. Consequently, the public health professional can best resolve the trade-off by choosing a campaign that targets randomly selected or resistant individuals. Moreover, the intervention can be relatively small, so long as people most amenable to change are not targeted. For instance, targeting only 20% of the population can trigger a large behavioural spillover. This reasoning may at first seem counterintuitive but, when everyone except the most resistant is likely to respond to the intervention, the best way to maximize behaviour change is to avoid targeting those most amenable to change. The direct effect of the intervention on randomly selected or resistant individuals sparks an indirect effect among those amenable to change, which then leads to further indirect effects as more and more people stop smoking in a cascade of behaviour change.

When the probability of responding to an intervention by changing behaviour is very low for everyone except those most amenable to change (Fig. 3), people already on the cusp of stopping smoking are likely to respond to the media campaign whereas most others are unlikely to respond. In this situation, targeting those amenable to change maximizes both the direct and total effects of the intervention and the public health professional can resolve the basic trade-off by choosing a campaign that targets people amenable to change. However, in this scenario the professional faces a larger challenge than determining how to resolve the trade-off between increasing the direct or indirect effect of the intervention. The situation is generally unfavourable for behaviour change and only people ready to stop smoking are likely to respond to the intervention. Therefore, although targeting people amenable to change results in more behaviour change than targeting randomly selected or resistant individuals, the primary problem is that the intervention is inadequate. The best approach, then, is to try to improve the intervention, which in turn may alter the best way to resolve the trade-off between its direct and indirect effects.

Faced with the typical situation in public health in which people have different attitudes and beliefs and where an intervention is likely to reach only a limited number, public health professionals must make an informed decision on who to target. The modelling results reported here show that the behaviour change achievable is strongly influenced by the heterogeneity of attitudes and beliefs common in many populations. However, professionals may not always be able to identify or measure that heterogeneity. For example, attitudes may not be observable and people may be unwilling to share their preferences openly when asked.^24^ In addition, budgetary and time constraints may make it impossible or impractical to conduct a representative survey of attitudes and preferences. In this context, targeting randomly selected individuals with the intervention can be a comparably safe bet because, in terms of the intervention’s effectiveness, targeting randomly selected individuals falls between targeting the extremes of amenable or resistant individuals, thereby avoiding the potential weaknesses of both (Fig. 2 and Fig. 3). The public health professional’s only task in such situations is to avoid biased selection. More detailed information on how direct and indirect effects are influenced by the probability of changing behaviour is available from the data repository.^23^

## Conformity and anticoordination incentives

The different approaches to changing smoking behaviour discussed above assume that the interventions involve incentives for people to coordinate their behaviour.^25^^,^^26^ In this setting and many similar situations which involve coordination incentives, the influences of conformity and coordination incentives act in the same direction. In other situations, however, conformity and anticoordination incentives can act as countervailing influences.

**Fig. 4:**
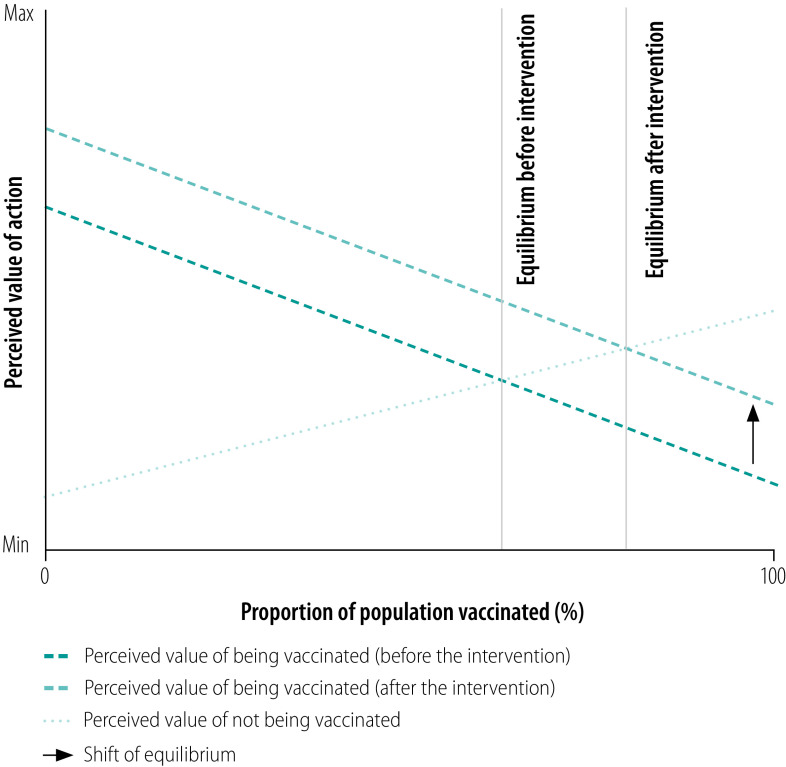
Population vaccination rate and the perceived value of vaccination in an anticoordination setting

Note: The perceived value of vaccination may decrease as the number of people vaccinated increases while the costs of vaccination remain unchanged. The benefit of vaccination can approach zero when nearly everyone in the population is vaccinated because the risk an unvaccinated individual will catch the disease is small. In contrast, the perceived value of not getting vaccinated increases as more people get vaccinated (i.e. the anticoordination incentive). The end result is a stable equilibrium with some people choosing vaccination and others not. An intervention can shift the equilibrium (arrow) by providing incentives, information or behavioural nudges or reducing costs and concerns about side-effects. More information about the intersection of value functions is available from the data repository.^33^

A review of the literature on vaccination behaviour illustrates this opposition between conformity and anticoordination incentives. A considerable amount of research has demonstrated the importance of social norms and conformity for vaccine delivery.^9^^,^^27^^–^^31^ Furthermore, conformity has been reported to be relatively efficient in supporting vaccination campaigns when coupled with an initial focus on well connected individuals.^32^ Public health professionals generally aim to increase the proportion of vaccinated individuals in the population beyond the threshold for herd immunity and social influence may support them in this endeavour. However, once a certain proportion has been vaccinated, anticoordination incentives can favour opting out of vaccination.^30^ For example, the perceived value of vaccination may decrease as the number of people vaccinated increases (Fig. 4) because the risk an unvaccinated person will catch the disease declines as vaccination becomes more widespread.^30^ In this context, general incentives for vaccination involve some pressure to do what others are not doing. With antismoking campaigns in contrast, the incentives involve persuading people to do what others are doing. Vaccination, then, can present people with an anticoordination incentive: a single unvaccinated person in a population in which everyone else has been vaccinated has little reason to get the vaccine. The same is probably true for two or even a dozen unvaccinated individuals. More detailed information on coordination incentives is available from the data repository.^33^

The question for public health professionals is, what proportion of the population must be vaccinated for the incentive to switch from favouring vaccination to favouring the decision not to vaccinate? The answer depends on how the net value of the vaccine varies with the number of people vaccinated. The perceived cost of contracting the disease when unvaccinated must be balanced against the possible fixed costs of vaccination associated, for example, with possible allergic reactions and the expense of, and time needed to go for, vaccination. In addition, there is the fear fanned by misinformation and antivaccine movements.^34^ Once enough people have been vaccinated to ensure that, for the unvaccinated, the perceived value of not getting the vaccine exceeds the perceived value of vaccination, the proportion of people vaccinated will reach a stable equilibrium. Put differently, unvaccinated people are reaping the benefit of the reduced infection risk associated with herd immunity without paying the cost of vaccination.^35^ However, if the equilibrium point is below the threshold for herd immunity, public health professionals will need to design interventions that increase the vaccination rate to a point above the herd immunity threshold (Fig. 4). Regardless of the precise benefits and costs of being vaccinated or not, anticoordination incentives, unlike coordination incentives, do not amplify the effects of an intervention via spillover behaviour. Indeed, they do exactly the opposite by encouraging some people not to adopt the health professional’s preferred behaviour.

To support the policy objective of achieving a vaccination rate above the threshold for herd immunity, public health professionals could use different strategies to counter anticoordination incentives. First, they can make vaccination rewarding: reduce the monetary and time cost of vaccination or provide financial incentives, for example, in the form of cash transfers, lottery tickets, vouchers or material goods.^36^^–^^39^ Second, they can make vaccination appealing: public health professionals should communicate effectively about the low risk of vaccination side-effects and the health benefits of the vaccine and counter people’s fears, which may originate from misinformation.^34^^,^^35^ Third, they can make vaccination easy: use behavioural nudges, for example in the form of reminders, prompts or default options. This could help to reduce the perceived time costs of vaccination and overcome inertia and a lack of motivation in people who are generally open to being vaccinated.^37^^,^^40^

A combination of these strategies is recommended,^37^ especially when mandates are not permissible or practical.^41^ However, not all strategies will have the desired effect in all settings. Direct financial incentives, for example, may reduce the overall level of vaccination through so-called crowding out:^42^^–^^45^ financial incentives could lessen the social motivation of some people who would get vaccinated primarily to protect vulnerable others. Offering payment can turn social behaviour into market behaviour, possibly leading people to abstain. Whatever the best solution is in a specific setting, public health professionals should focus on countering anticoordination incentives to promote large-scale vaccination uptake beyond the threshold of herd immunity.

## Conclusion

When public health professionals implement behavioural change interventions that rely on social influence, they can expect results ranging from the spectacular to the negligible. Although specific mechanisms such as conformity and coordination incentives can dramatically amplify the beneficial effects of a policy initiative, the details are crucial and the policy’s success is influenced by several poorly understood and subtle mechanisms.^45^^,^^46^ Practical recommendations for maximizing behaviour change based on the concepts discussed are summarized in Box 1.

Box 1 **Practical recommendations for maximizing behaviour change**

**1. Are the resources and infrastructure available to reach the whole population with a behaviour change intervention?**

- Yes: behavioural spillover can be cost-efficient but may not be necessary to achieve the policy goal.
- No: behavioural spillover might help to indirectly reach those who cannot be reached directly (the following steps explain how this could be achieved).

**2. Is detailed information available about the heterogeneity of attitudes in the population?**

- Yes: knowledge about attitudes can be used to maximize the total effect of the intervention, not just the direct effect (the following step explains how this could be achieved).
- No: there are different solutions: (i) develop the methods and capacity needed to assess existing attitudes; (ii) use available sociodemographic data as a proxy for attitudes;^47^ or (iii) choose to target randomly selected individuals with the intervention because this a relatively safe option that will avoid the pitfalls of designing an intervention to target either amenable or resistant individuals when the predominant attitudes of the population are unknown.

**3. How effective is the intervention in changing behaviour among different individuals in the population?**

- If it is effective among everyone except the most resistant, it is advisable to avoid targeting the most amenable.
- If it is effective among only the most amenable, the campaign is not particularly effective overall. Solutions: (i) improve the effectiveness of the intervention; (ii) selectively target only the small amenable population using low-cost measures; (iii) directly target a large subset of the population with an intervention (in many cases, this will be much more than 50%); or (iv) design an intervention that decouples group identity from the target behaviour if resistance to the behaviour change is related to deeply rooted group identities or traditions.^13^

**4. Does the target behaviour involve coordination incentives (i.e. individuals will prefer behaviour A to behaviour B when everyone else is exhibiting behaviour A and will prefer behaviour B to behaviour A when nobody else is exhibiting behaviour A)?**

- Yes: public health professionals should focus on triggering behavioural spillover because conformity and coordination incentives will both support the policy objective once the desired behaviour is sufficiently common.
- No: it is possible that anticoordination incentives may be encouraging people to do the opposite to others. These can be attenuated by: (i) increasing the value of the target behaviour (e.g. through financial incentives); (ii) providing information; or (iii) employing behavioural nudges.

**5. Evaluating the impact of the behavioural change intervention**

- Even if an intervention seems unsuccessful at first, it may create large-scale behavioural change through spillover. Consequently, the effect of the intervention should be evaluated in a sample of people who were not targeted. Repeated evaluations are advisable as a single evaluation immediately after the intervention ends could miss indirect effects that unfold over time.

We hope our observations will contribute to the field of public health by illustrating how the possible effects of behavioural change interventions can vary according to the people’s different attitudes and beliefs. As a result, these interventions can have unexpected, unhelpful and counterintuitive consequences. Ideally, public health professionals should gain a better understanding of both the direct and indirect effects of their interventions by assessing the different attitudes and beliefs among people in their target population. The strategies we describe could help public health professionals take their first steps towards effectively managing behavioural spillover when designing public health interventions.
